# Exploring Gender Differences in Volunteer Behavior Among Older Asian Americans

**DOI:** 10.1093/geroni/igab046.086

**Published:** 2021-12-17

**Authors:** Patrick Ho Lam Lai, Christina Matz, Cal Halvorsen

**Affiliations:** 1 Boston College School of Social Work, Chestnut Hill, Massachusetts, United States; 2 Boston College, Chestnut Hill, Massachusetts, United States

## Abstract

Prior research has documented the health and well-being effects of volunteerism in later life, and that positive outcomes increase in the first 100 volunteer hours/year and slightly increase between 100-200 hours. Given this, it seems that using an intersectional lens to explore disparities in volunteer behaviors and what might explain them is important from a health equity standpoint. Using data from 268,194 individuals aged 65-85 from the most recently available Volunteer Supplement of the Current Population Survey, this study found that White older adults were most likely to spend any time in volunteer activities, while Asian and Hispanic older adults were least, across all racial/ethnic groups. Further, the percentage of older Asian women volunteering in the 100-200 hour range (27%) was significantly higher than that of older Asian men (19%). Social and cultural factors that may explain these racial/gender differences and implications for recruiting older adults as volunteers are discussed.

